# Landscape of official development assistance for nutrition data and information systems

**DOI:** 10.1136/bmjgh-2021-007370

**Published:** 2022-03-08

**Authors:** Alexandra Farina, Yashodhara Rana, Augustin Flory, Kyle Borces, Caroline Snead, Rebecca Heidkamp

**Affiliations:** 1 Results for Development Institute, Washington, District of Columbia, USA; 2 ThinkWell, Washington, District of Columbia, USA; 3 Independent Researcher, Los Angeles, California, USA; 4 International Health, Johns Hopkins University Bloomberg School of Public Health, Baltimore, Maryland, USA

**Keywords:** nutrition

## Abstract

Nutrition data and information systems (ND&IS) are critical to guide the prioritisation, collection, analysis and dissemination of nutrition data in countries. However, there is limited guidance for countries regarding how to invest in their ND&IS and little is known about current financing allocations by both countries and donors. This hinders our ability to identify the most critical funding gaps and to effectively advocate for increased financial commitments to ND&IS. To better characterise donor investments, we conducted a review of Official Development Assistance (ODA) financing for ND&IS between the years 2017 and 2019. The analysis showed overall donor financing for ND&IS is not trending up between 2017 and 2019 with the majority of funding being channelled through multilateral organisations to the health sector and spent on global initiatives and emergency early warning system and surveillance activities. Given these findings, donors should dedicate at least 5% (4%–6%) of nutrition investments, alongside country governments, to support country capacity building and strengthening of ND&IS. Donors should also consider channelling a larger part of ODA for ND&IS activities through public institutions to build their capacity to manage ND&IS strengthening.

**Summary box**

- Nutrition data and information systems are designed to collect, analyse and share timely nutrition data to monitor the status of nutrition priorities and programmes at the national level and inform decisions around programme planning, budgeting and advocacy.
- There are no accessible estimates of current financing allocations for nutrition data and information systems (ND&IS) by both countries and donors.
- Overall annual Official Development Assistance (ODA) for ND&IS, while significant at an average of $47 million (lower estimate) and $89 million (upper estimate) per year, is not trending up, with an increase between 2017 and 2018 and an equivalent decrease between 2018 and 2019.
- Most funding was directed to global initiatives, early warning system/surveillance systems, and periodical data collection activities, and flowed through multilateral organisations and non-governmental organisations. Comparatively, less funding was spent on country capacity strengthening activities or channelled directly to governments and public sector institutions.
- The top ND&IS donors are the same as the top nutrition financing donors, and most of their ND&IS development assistance is channelled to the health sector with less to other sectors addressing underlying causes of malnutrition.
- Donors should dedicate at least 5% of nutrition investments, alongside country governments, to support country capacity building and strengthening of ND&IS.
- Donors should also consider channelling a larger part of ODA for ND&IS activities through public institutions to build their capacity to manage ND&IS strengthening.

## Introduction

“Without good data, we are flying blind. If you can’t see it, you can’t solve it,” as the late Kofi Annan famously said about nutrition.

Highlighting the vast gaps and weaknesses in nutrition data, the Global Nutrition Report (GNR) called in 2014 for a data revolution to accelerate progress towards the World Health Assembly (WHA) targets and support the achievement of the Sustainable Development Goals. In 2017, the GNR used the concept of the data value chain (DVC)^1^ as a framework to guide improvements in data availability and use.^2^ Since the introduction of this framework, significant work has been done at the global and country levels to strengthen the DVC and country data plans, however the COVID-19 pandemic has highlighted the urgent need to increase investments in data and information systems to provide an effective response to the challenges brought about by the pandemic.

**Figure 1:**
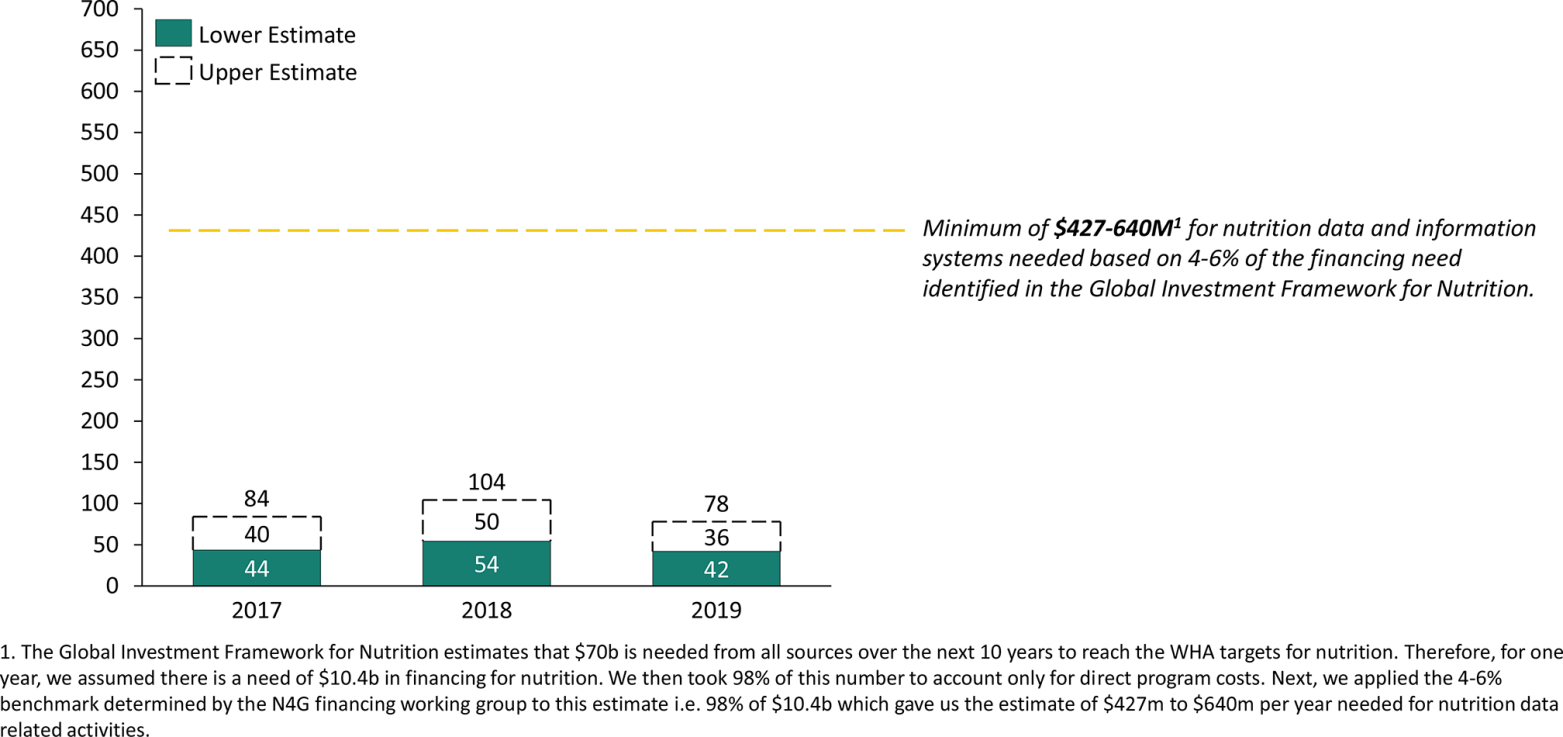
Total spending towards ND&IS in 2017–2019, millions US dollars.

Nutrition data and information systems (ND&IS) are an integrated set of principles, practices and processes guiding the prioritisation, collection, storage, organisation, analysis and dissemination of essential nutrition-related data drawn from multiple sectors and sources. ND&IS are critical to provide timely data to monitor the status of national and subnational nutrition priorities and programmes to inform decisions around programme planning, budgeting and design. However, there are no accessible estimates of the financing needs to strengthen global and country ND&IS. The 2020 Nutrition for Growth (N4G) Financing for Data Thematic Working Group concluded that a benchmark of 4%–6% of funding for country multisectoral nutrition plans should be allocated to data-related activities (Ellen Piwoz on behalf of the Financing for Data Thematic Working Group, 2019). This corresponds to $427–$640 million annually based on the Global Investment Framework for Nutrition (GIFN) costing estimates to achieve the WHA global nutrition targets (see figure 1 for a breakdown of this estimate).^3^ However, these estimates are only focused on a limited set of direct nutrition interventions primarily delivered by the health sector, and the required ND&IS investments in data are much larger when we account for multisectoral nutrition plans.

How close to these benchmarks are current investments in ND&IS? There is little known about current financing allocations to ND&IS by both countries and donors. This limits our ability to identify the most critical funding gaps and to effectively advocate for new strategic investments in ND&IS.

The goal of this paper is to present an aggregate picture of Official Development Assistance (ODA) for ND&IS to help inform the donor community’s strategic decisions and coordination as well as country perspectives on their priorities. This is timely given the upcoming 2021 N4G Summit which will provide an opportunity for the nutrition community to renew and expand commitments towards improving malnutrition globally. It might also be of interest to national governments, implementing partners and advocates in their efforts to support ND&IS.

## How donor financing for ND&IS was assessed

**Figure 2:**
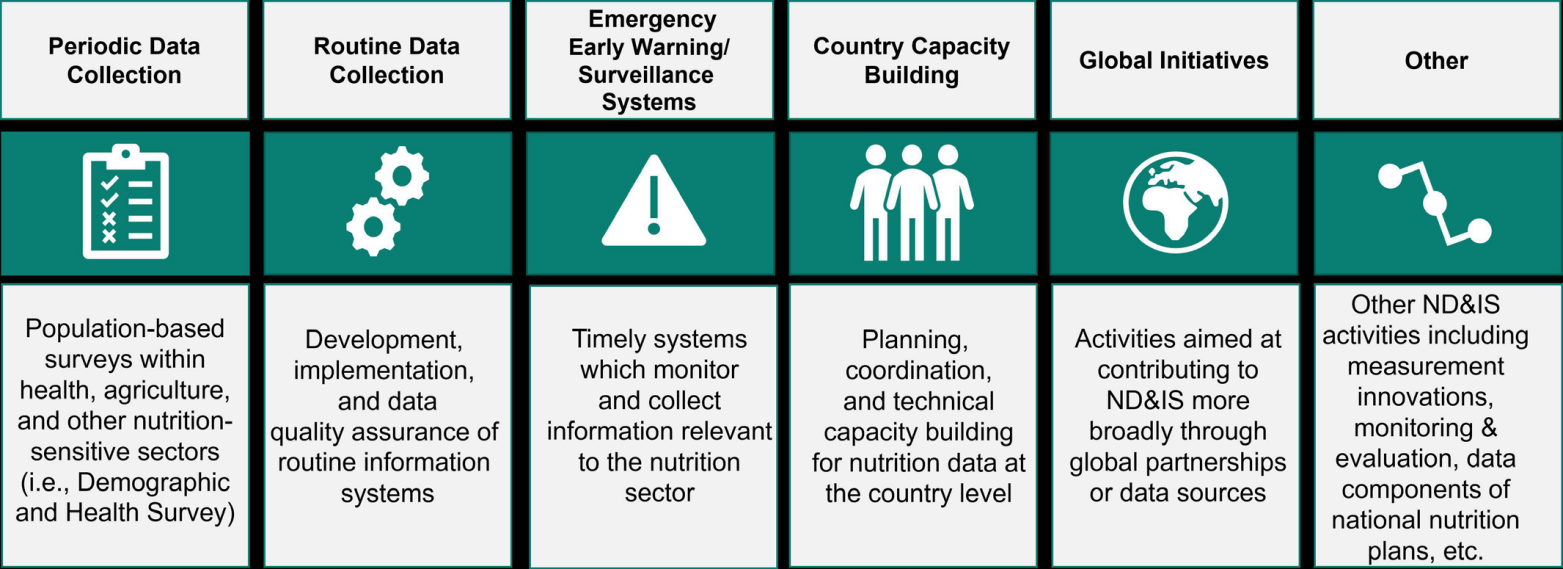
Description of ND&IS core activities.

This analysis focuses on core ND&IS activities while recognising that data and information strengthening in other sectors as well as broader statistical capacity building including international initiatives to support data for development have downstream benefits for ND&IS. We considered the six domains defined in figure 2 to be ND&IS core activities. These are adapted from an earlier framework^4^ on the major costs behind ND&IS. Please note this analysis does not capture funding flows to independent evaluators. While such investments are critical for informing nutrition policies and programmes, they tend to be programme specific and are not building blocks of ND&IS at the country level. A detailed description of the data source and analytical methods used is provided in box 1.

Box 1 **Description of data source, analytical methods and limitations**

- *Data source*

We compiled our dataset by first extracting disbursement data from the Organization for Economic Co-operation and Development (OECD) Creditor Reporting System (CRS) database* from the years 2017–2019, that is, the most recent years for which data are available (see online supplemental box S1 for more information on the CRS database).

- *Methodology*

As a first step, for each year of data, we used a keyword search to identify disbursements across sectors relevant to nutrition data and information systems (ND&IS) (refer to online supplemental tables S1–S3 for the keywords used). Next, we manually screened each transaction to determine if the project included ND&IS core activities. Each disbursement determined to be ND&IS relevant was then tagged as a lower or upper estimate. Lower estimate tagging was used when the full share of the disbursement was considered relevant for ND&IS while disbursements were tagged as an upper estimate if ND&IS activities were one of several objectives and the exact share of the disbursement could not be unpacked. For the lower estimate, we captured the full disbursement value, while for the upper estimate we discounted the full disbursement value by 50% since the identified ND&IS activity contributed towards one of several objectives alongside other activities. In addition, we classified each disbursement under the most applicable ND&IS category specified in figure 2. In the few cases where disbursements had more than one explicit ND&IS activity, the disbursement was split evenly across ND&IS categories (see online supplemental figure S1 for more details).

Given that ND&IS benefits from broader investments in health information systems, the team also captured disbursements on health management information systems and District Health Information Software 2 using keywords found in online supplemental table S2.

- *Limitations*

Several limitations should be noted. First, there is no purpose code for ‘information systems’ for nutrition so we relied on a targeted keyword search to compile our dataset. Next, within our dataset, many disbursements included multiple objectives and provided limited information to specify the ND&IS activities. To account for these limitations, we included upper estimates to reflect the uncertainty about the value of ND&IS activities. The upper estimate was discounted by 50% to reflect that there was at least one other objective mentioned in a disbursement. We recognise this is an overestimate since a transaction can have more than two objectives and the dollar amounts going towards the each of these objectives may not be equally split. Therefore, we conducted a sensitivity analysis to determine how the upper estimate would change if it was discounted by 25% and 75% instead of 50%. We found in all scenarios, the total spending for ND&IS remains below the minimum financing need for ND&IS recommended by the Investment Framework for Nutrition (see online supplemental figure S2 for specific results).

Additionally, our analysis does not include data on philanthropic contributions or other private sources of funding, with a few noted exceptions (Bill and Melinda Gates Foundation and Children’s Investment Fund Foundation), as these data are not available in the CRS. Lastly, large nutrition donors such as UNICEF and the World Bank which provide country-level technical assistance for ND&IS alongside their large programmes may not be accurately represented in our estimates given these activities are not always explicitly captured in the CRS project descriptions.

*OECD. ‘Creditor Reporting System.’ OECD International Development Statistics, 2019. https://doi.org/10.1787/data-00061-en (accessed August 2020).

10.1136/bmjgh-2021-007370.supp2 Supplementary data

10.1136/bmjgh-2021-007370.supp4 Supplementary data

10.1136/bmjgh-2021-007370.supp5 Supplementary data

10.1136/bmjgh-2021-007370.supp6 Supplementary data

10.1136/bmjgh-2021-007370.supp1 Supplementary data

10.1136/bmjgh-2021-007370.supp3 Supplementary data

## Overall spending on ND&IS

Overall, ODA for ND&IS, while significant at an average of $47 million (lower estimate) and $89 million (upper estimate) per year, is not trending up, with an increase between 2017 and 2018 and an equivalent decrease between 2018 and 2019 (figure 1). For calibration purposes, the lower estimate of donor funding for ND&IS in 2017 ($44 million) corresponds to 3.1% of the $1397 million disbursed in 2017^5^ towards nutrition-specific priority interventions specified in the GIFN, which is significant. However, spending is not trending in the right direction, particularly given that the GIFN calls for a rapid increase in donor investments year on year.

## What types of activities were funded?

**Figure 3:**
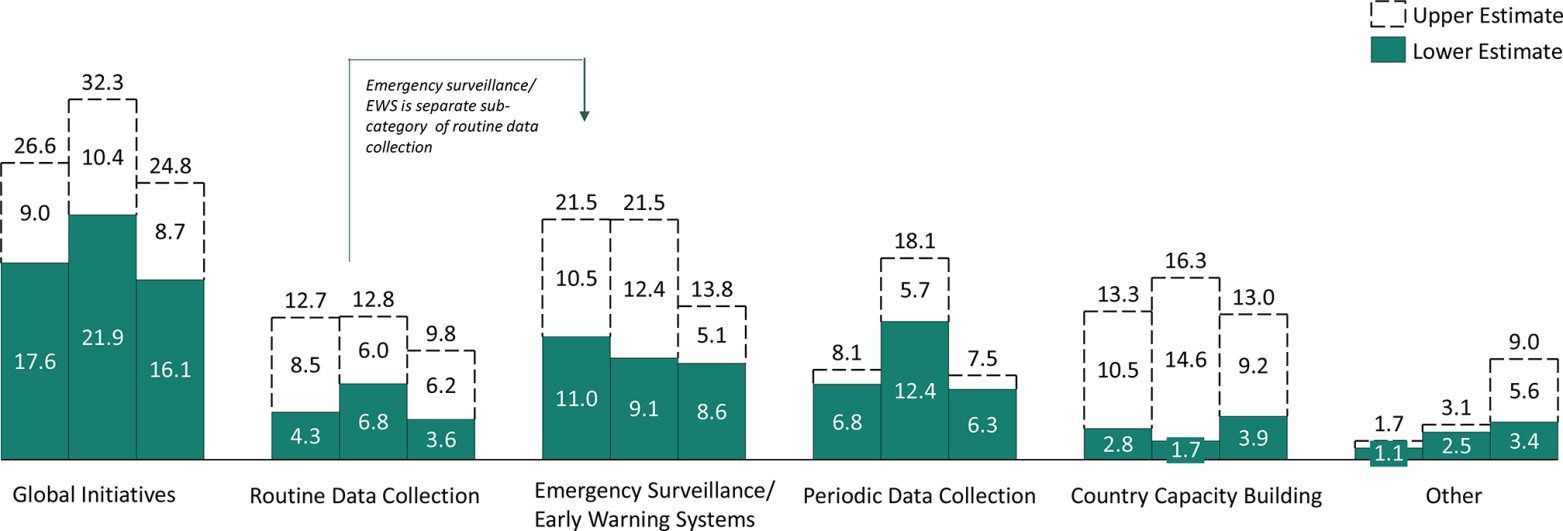
Breakdown of spending by ND&IS component in 2017–2019, millions US dollars.

Based on the lower estimate, the majority of funding was spent on global initiatives followed by early warning system (EWS)/surveillance systems and periodical data collection (figure 3). Examples of global initiatives included the National Information Platforms for Nutrition, Maximizing the Quality of Scaling Up Nutrition Plus (MQSUN+), Alive & Thrive and the WHO monitoring of progress towards the global nutrition targets. It is important to note that global initiatives may also have provided some country-level technical assistance. Within the early warning and surveillance system category, significant funding went towards the Famine Early Warning Systems Network and projects focused on establishing an emergency food security or nutrition surveillance/EWS. Periodical data collection included household surveys such as the Demographic and Health Survey, the Living Standards Measurement Study, and Standardized Monitoring and Assessment of Relief and Transitions surveys.

Comparatively, less funding was identified for routine data collection and country capacity building activities, although capacity building is likely under-reported as it is often bundled with the other ND&IS components. Routine data collection included investments for nutrition information systems strengthening. While the District Health Information Software 2 (DHIS2) is used for nutrition, we did not include it here since there were no disbursements that aimed to specifically integrate nutrition indicators into the DHIS2 platform. The country capacity building category captured activities related to strengthening the collection, analysis, use, quality and monitoring of nutrition-related data. Investment in country capacity building should remain a priority since ND&IS are primarily country owned and driven, and therefore building the capacity of government and local partners to strengthen and operate the system is critical for effectiveness and sustainability (see online supplemental table S4 for additional examples of the projects captured under each category).

10.1136/bmjgh-2021-007370.supp7 Supplementary data

## What channels did funding flow through and to where did it go?

**Figure 4:**
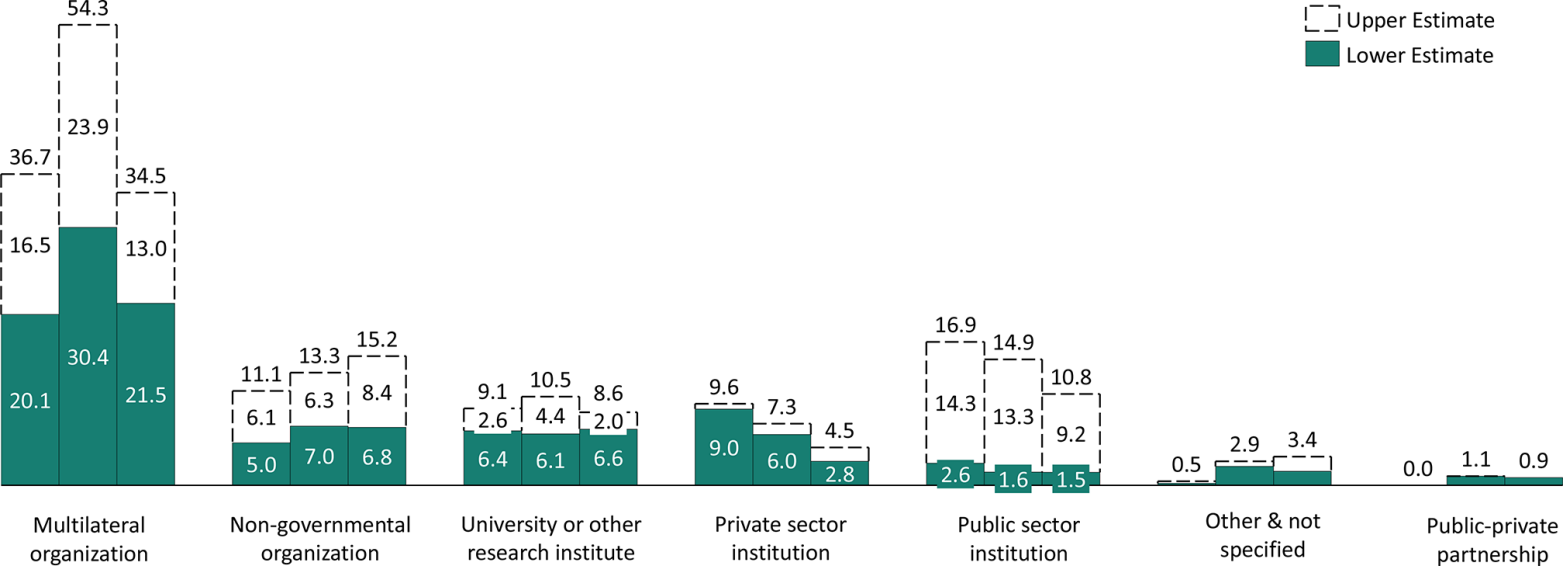
Breakdown of spending by channel type in 2017–2019, millions US dollars.

Most of the funds for ND&IS flowed through multilateral organisations, specifically UNICEF, World Food Programme, and Food and Agriculture Organization (figure 4). The next highest levels are through non-governmental organisations and universities/research institutes. Very little ODA captured in the Creditor Reporting System (CRS) went directly towards governments and public sector institutions, with that share decreasing over time. While multilaterals are critical players in the nutrition landscape and often work at the country level with and through local partners, it is critical that more funding flows directly to the country governments to build their capacity to manage ND&IS strengthening activities.

To assess the sector towards which each contribution will go, each transaction is assigned a purpose code within the CRS database (see online supplemental box S1 for specific purpose code definitions). The majority of ND&IS disbursements come from the ‘basic nutrition’ purpose code which is a subcode of ‘health’. There was notably less spending from the food assistance and agricultural policy and administrative management purpose codes. Given nutrition is multisectoral in nature, these findings highlight a potential gap in ND&IS relevant activities outside of the health sector.

We also captured spending focused on general health management information systems (HMIS) and DHIS2 activities given spending in these areas can provide downstream benefits for ND&IS since nutrition-specific programmes are mainly delivered through health systems. At an average of around $4.3 million per year between 2017 and 2019, this amount is relatively small, however there may be significantly more donor spending on HMIS and DHIS2 that our methodology is not capturing given limited project descriptions. Activities captured included implementation and/or training of HMIS/DHIS2 systems.

## Who were the top donors?

The European Union Institutions disbursed the most funding to ND&IS activities across all 3 years, followed by the Bill and Melinda Gates Foundation, the UK and the USA. These top donors for ND&IS are mostly consistent with the top donors of overall ODA for nutrition.^6^

## Conclusion

ND&IS are critical to enable countries to make evidence-based decisions around nutrition programme development, resource mobilisation and policies. Based on our findings of the current financial landscape of ND&IS, we propose six recommendations for donors:

First, donors should work with governments and partners in each country to strengthen coordination and sharing of information on ND&IS to identify gaps and priorities across the nutrition data value chain, especially given pressures on domestic and donor budgets due to the COVID-19 pandemic. Governments and partners, including donors, may want to consider creating country-level technical working groups on ND&IS, if not already existing, to encourage and actively participate in collaboration, exchange of information and more strategic approaches.

Second, donors should provide financial and technical support to countries to develop and cost strategic data and information systems plans (including considerations for their financing), focusing on the different stages of the nutrition data value chain. Previous research which reviewed costed national nutrition plans for 58 Scaling up Nutrition (SUN) countries found that fewer than half of the countries had costed plans with data and monitoring and evaluation sections.^4^ While MQSUN+ and other SUN partners have supported countries in developing and costing nutrition data plans, their initiatives are coming to an end and so it is critical to find new ways to continue supporting countries moving forward including through SUN 3.0.

Third, donors should dedicate at least 5% (4%–6%) of nutrition investments, alongside country governments, to support country capacity building and strengthening of ND&IS. Donors should also consider channelling a larger part of ODA towards ND&IS activities through public institutions to increase ownership and sustainability and build country capacity to manage ND&IS. As part of this, we encourage the GNR to use the Nutrition Accountability Framework to hold both donors and governments accountable for strengthening data systems and capacities across the data value chain and supporting the use of data for decision-making.^7^

Fourth, donors should support the integration of more nutrition service delivery indicators within the health information system. This can be a cost-effective way of improving nutrition data since many nutrition-specific programmes are mainly delivered through health system platforms. In addition, support for data and information systems in other sectors such as social protection should be prioritised to monitor relevant nutrition indicators.

Fifth, donors should improve reporting within the CRS to track funding more accurately towards ND&IS including more consistent capture of ND&IS activities in project descriptions.

Lastly, donors should consider supporting further research on the costing and financing of ND&IS to improve understanding of the key gaps and challenges and the development of practical solutions.

## Data Availability

[Dataset] OECD. 'Creditor Reporting System.' OECD International Development Statistics, 2019. https://doi.org/10.1787/data-00061-en (accessed August 2020). Data are available upon request
