# Supplementary material for: Landscape of official development assistance for nutrition data and information systems
Source: BMJ Glob Health. 2022 Mar 8;7(3):e007370. doi: 10.1136/bmjgh-2021-007370 (PMC8905917; doi:10.1136/bmjgh-2021-007370)
Supplement: Supplementary data [file bmjgh-2021-007370supp002.pdf]

Supplemental Box 1: Overview of OECD Creditor Reporting System

The **Creditor Reporting System** is a database which includes transaction-level data for all Development Assistance Committee (DAC) country donors, multilateral donors, and private grants. The database includes information which can be used to understand where aid goes, what purpose it serves and what policies it aims to implement.

Overview of how OECD CRS data are captured and tagged:

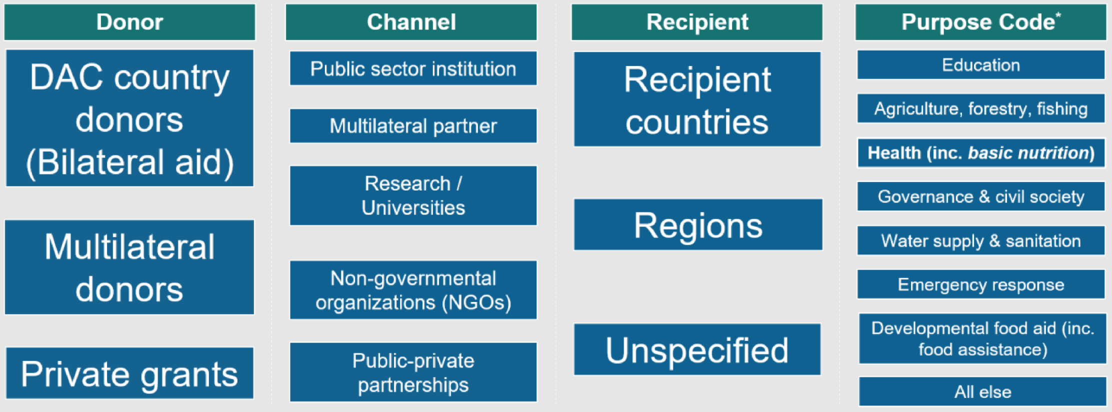

**The purpose code is used to identify the sector of destination of a contribution.*

Definitions of Top Purpose Codes:

1. **Basic nutrition**: Interventions related to micronutrient deficiency identification and supplementation; infant and young child feeding promotion including exclusive breastfeeding; non-emergency management of acute malnutrition and other targeted feeding programs (including complementary feeding); staple food fortification including salt iodization; nutritional status monitoring and national nutrition surveillance; research, capacity building, policy development, monitoring and evaluation in support of these interventions.
2. **Food assistance** - Supply of edible human food under national or international programmes including transport costs, cash payments made for food supplies; project food assistance aid and food assistance aid for market sales when benefiting sector not specified.
3. **Agriculture policy and administrative management** - Agricultural sector policy, planning and programmes; aid to agricultural ministries; institution capacity building and advice; unspecified agriculture.
4. **Health policy and administrative management** - Health sector policy, planning and programmes; aid to health ministries, public health administration; institution capacity building and advice; medical insurance programmes; including health system strengthening and health governance.
5. **Material relief assistance and services** - Shelter, water, sanitation, education, health services including supply of medicines and malnutrition management, including medical nutrition management; supply of other nonfood relief items (including cash and voucher delivery modalities) for the benefit of crisis-affected people.
