# Supplementary material for: Landscape of official development assistance for nutrition data and information systems
Source: BMJ Glob Health. 2022 Mar 8;7(3):e007370. doi: 10.1136/bmjgh-2021-007370 (PMC8905917; doi:10.1136/bmjgh-2021-007370)
Supplement: Supplementary data [file bmjgh-2021-007370supp004.pdf]

Supplemental Table 1: Nutrition-specific Keywords

We extracted disbursements related to ND&IS by first pulling all nutrition-related projects in the CRS. These keywords were taken from the keyword list developed for Results for Development's work on Tracking Aid for the World Health Assembly Nutrition Targets[1]. Keywords used multiple languages, including English, French and Spanish.

Nutrition keywords	
ALNP	infant and young child feeding
ANJE	infant growth
ATLC	insuffisance pondérale
ATLU	intrauterine growth restriction
ATPE	iodation du sel
BMI	iodiz
CMAM	lactancia
IMC	lactat
iron	lait maternel
IUGR	leche materna
IYCF	légume
MUAC	linear growth
RCIU	low birth weight
RUTF	low birthweight
zinc	low sodium
aliment thérapeutique	malnourish
alimentación complementario	mid-upper arm circumference
alimentación del lactante y del niño pequeño	näring
alimentation complémentaire	nourish
alimentation du nourrisson et du jeune enfant	nourr
alimentos complementarios	nutri
alimentos terapéuticos listos para consumir	obesidad
alimentos terapéuticos listos para usar	obésité
aliments complémentaires	obesity
aliments transformés	orange fleshed sweet potato
allaitement	orange-fleshed sweet potato
alto contenido de azucar	overweight
alto contenido de grasa	patate douce à chair orange

[1] D'Alimonte M, Thacher E, Clift J, *et al.* Tracking aid for the WHA nutrition targets: Progress towards the global nutrition goals between 2015 to 2017. Washington, D.C.: Results for Development 2018. https://r4d.org/resources/tracking-aid-wha-nutrition-targets-global-spending-roadmap-better-data/ (accessed 17 May 2021).

amamant	perímetro braquial
anaemia	peso para la edad
anemia	peso para la estatura
anémie	poids pour l'âge
arroz dorado	poids pour taille debout
azucaradas	poids-pour-l'âge
azucarados	poids-pour-taille debout
bajo contenido en sodio	poids-taille
bajo peso al nacer	processed food
bioenriquecimiento	protein energy
biofort	ravinto
body mass index	ravitsemus
breast-	RCIU
breast milk	ready to use therapeutic food
breastfeeding	ready-to-use therapeutic food
breastmilk	reduce sodium
camote anaranjado	reducir el consumo de sal
camote de pulpa anaranjada	reducir sodio
circonférence du bras à mi-hauteur	réduction de sel
comida precesada	réduire le sodium
complementary food	restricción del crecimiento intrauterino
consommation de sel	retard de croissance
consommation de sucre	retraso en talla
consumo de azúcar	riche en graisse
consumo de sal	riz doré
crecimiento fetal	sal yodada
crecimiento infantíl	salado
crecimiento lineal	salé
croissance chez l'enfant	salt intake
croissance de l'enfant	salt reduction
croissance infantile	salty
croissance linéaire	SAM treatment
debout pour l'âge	sel iodé
debout-pour-l'âge	sobrepeso
dietary diversity	stunting
diversité alimentaire	sucré
emaciación	sugar consumption
émaciation	sugar-sweeten
enriquecimiento	sugary
ernæring	suplementos de hierro
Ernährung	supplémentation en fer

estatura para el peso	surpoids
estatura para la edad	taille pour l’age
fetal growth	táplálás
folic	teneur en sodium
fólico	trans fat
folique	trans-fat
fortif	under weight
golden rice	undernourish
gras trans	underweight
grasas trans	under-weight
growth monitoring	vitamin
HarvestPlus	voeding
height-for	výživa
high in fat	wasting
índice de masa corporal	weight-for
indice de masse corporelle	
