# Supplementary material for: Landscape of official development assistance for nutrition data and information systems
Source: BMJ Glob Health. 2022 Mar 8;7(3):e007370. doi: 10.1136/bmjgh-2021-007370 (PMC8905917; doi:10.1136/bmjgh-2021-007370)
Supplement: Supplementary data [file bmjgh-2021-007370supp005.pdf]

## Supplemental Table 2: Nutrition Data Keywords

Within the catchment of nutrition projects extracted using keywords from **Supplemental Table 1** (nutrition-specific keywords), we used nutrition data keywords to filter for nutrition data-related activities only.

| Nutrition data keywords | |
|---|---|
| Access to Nutrition Index | International Dietary Data Expansion |
| Agriculture management information system | Joint Child Malnutrition Estimates |
| Biometric data | Knowledge for Nutrition (K4N) |
| Cadré intégré de classification de la sécurité alimentaire | La situation des enfants dans le monde |
| Calidad de fortificación | L'apport alimentaire |
| Clasificación integrada en fases de la seguridad alimentaria | Las Plataformas Nacionales de Información sobre Nutrición |
| Clúster Mundial de Nutrición | Le projet international d'expansion des données alimentaires |
| Community-based early warning system | Le renforcement de la nutrition |
| Comprehensive Food Security and Vulnerability Assessments | Living standards measurement |
| Compte à rebours jusqu'en 2030 | Medición de los niveles de vida |
| Countdown to 2030 | mHealth |
| Crop and Food Security Assessment Mission | Micronutrient and food consumption survey |
| Cuenta Regresiva al 2030 | MICS survey |
| Data for Decisions to Expand Nutrition Transformation | mNutrición |
| DATA-DENT | mNutrition |
| Datos abiertos nutricionales | m-Nutrition |
| Datos biométricos | Monitoreo y evaluación (M&E) de la nutrición |
| Datos de fortificación | Mouvement pour le renforcement de la nutrition |
| Datos de seguridad alimentaria | Mouvement SUN |
| Datos nutricionales | Movimiento SUN |
| Datos para tomar decisiones para expandir La transformacion nutricional | Movimiento SUN |
| Demographic and health survey | mSalud |
| Demographic health survey | mSanté |
| Demographic surveillance | Multiple indicator cluster |
| Dépistage nutritionnel | mVAM monitoring |
| DHIS2 | National Evaluation Platform |
| DHIS-2 | National family health survey |
| Dietary intake | National Information Platforms for Nutrition |
| District health information system | National nutrition survey |
| Données biométriques | NFHS |

| | |
|---|---|
| Données de fortification | NIPN |
| Données nutritionnelles | Niveau de vie |
| Données ouvertes nutritionnelles | Nutrition dashboard |
| Données pour la nutrition pour étendre la transformation de la nutrition | Nutrition data |
| Données sur la sécurité alimentaire | Nutrition information system |
| Échange global de données de fortification | Nutrition M&E |
| Education management information system | Nutrition monitoring |
| eHealth | Nutrition officer |
| El Intercambio de Datos | Nutrition screening |
| El Intercambio Global de Datos de Fortificación | Nutrition surveillance |
| Emergency Food Security Assessment | Nutrition surveillance system |
| Encuesta de consumo de alimentos | Nutrition survey |
| Encuesta de consumo de alimentos y micronutrientes | Nutrition system strengthening |
| Encuesta de Demografía y Salud | Nutritional open data |
| Encuesta de fortificación | Officier de nutrition |
| Encuesta de seguimiento del gasto público | Oficial de nutrición |
| Encuesta MICS | Outil de dissémination de données individuelles de consommation alimentaire au niveau mondial |
| Encuesta nacional de ingresos y gastos de los hogares | Partenariat mondial pour les données de développement durable |
| Encuesta nacional de nutrición | Performance Monitoring and Accountability |
| Encuesta nacional de salud familiar | Plataforma Nacional de Evaluación |
| Encuesta nutricional | Plateforme nationale d'évaluation |
| Encuesta SMART | Plateformes nationales d'information pour la nutrition |
| Enquête de consommation alimentaire | PMA2020 |
| Enquête de fortification | PNIN |
| Enquête de la nutrition | Pouls mondial |
| Enquête de suivi des dépenses publiques | Public expenditure review |
| Enquête démographique et sanitaire | Public expenditure tracking survey |
| Enquête nationale sur la santé de la famille | Pulso Mundial |
| Enquête nutritionnelle | Qualité de la fortification |
| Enquête par grappes à indicateurs multiples | Rapport mondial sur la nutrition |
| Enquête SMART | Renforcement des systèmes nutritionnels |
| Enquête sur les dépenses des ménages | Revisión del gasto público |
| Enquête sur les micronutriments et la consommation alimentaire | Revue des dépenses publiques |
| eSalud | Scaling Up Nutrition (SUN) |
| e-Santé | Scaling Up Nutrition Movement |
| Estado Mundial de la Infancia | seguimiento del desempeño y responsabilidad |

| | |
|---|---|
| Evaluación de la seguridad alimentaria | Seguimiento nutricional |
| Évaluation de la sécurité alimentaire | Service Provision Assessment |
| Examen de nutrición | Sistema de alerta temprana basado en la comunidad |
| Expansión de datos dietéticos internacionales | Sistema de alerta temprana de hambrunas |
| Famine early warning system | Sistema de información de gestión agrícola |
| FEWS-NET | Sistema de información de gestión educativa |
| Fomento de la Nutrición (SUN) | Sistema de información de gestión sanitaria |
| Food consumption survey | Sistema de información de salud |
| Food security assessment | Sistema de información sanitaria |
| Food security data | Sistema de vigilancia nutricional |
| Food security monitoring | Sistemas de información de nutrición |
| Food security monitoring system | Sistemas de vigilancia de seguridad alimentaria |
| Food Security Monitoring System | SMART survey |
| Fortalecimiento del sistema de nutrición | Standard de vie |
| Fortification data | State of Food Security and Nutrition in the World |
| Fortification quality | State of the World's Children |
| Fortification survey | Suivi de la sécurité alimentaire |
| GFDx | Suivi et évaluation nutritionnels |
| Global Fortification Data Exchange | Suivi nutritionnel |
| Global Individual Food consumption data tool | SUN Movement |
| Global Individual Food Consumption Data Tool | Surveillance de la sécurité alimentaire |
| Global nutrition cluster | Surveillance démographique |
| Global Nutrition Report | Surveillance nutritionnelle |
| Global Partnership for Sustainable Development Data | Système d'alerte précoce |
| Global Pulse | Système de surveillance nutritionnelle |
| GODAN | Système d'information de la santé |
| GPSDD | Système d'information et de gestion |
| Grappes à indicateurs multiples | Système d'information et de gestion de l'agriculture |
| Health information system | Système d'information et de gestion de l'éducation |
| Health management information system | Système d'information nutritionnelle |
| Herramienta global para la divulgación de datos sobre el consumo individual de alimentos | Système d'information sanitaire de district |
| Household Income and Expenditure Survey | Système d'information sur la gestion de la santé |
| Hunger and Nutrition Commitment Index | Tableau de bord nutrition |
| Index d'accès à la nutrition | Tablero de nutrición |

| | |
|---|---|
| Indicadores Múltiples por Conglomerados | Transform Nutrition |
| Índice de Acceso a la Nutrición | Vigilancia de la seguridad alimentaria |
| Informe de Nutrición Mundial | Vigilancia demográfica |
| Ingesta dietética | Vigilancia nutricional |
| Integrated Food Security Phase Classification | World Bank Nutrition Country Profiles |
