# Supplementary material for: Landscape of official development assistance for nutrition data and information systems
Source: BMJ Glob Health. 2022 Mar 8;7(3):e007370. doi: 10.1136/bmjgh-2021-007370 (PMC8905917; doi:10.1136/bmjgh-2021-007370)
Supplement: Supplementary data [file bmjgh-2021-007370supp006.pdf]

Supplemental Table 3: Nutrition-sensitive Keywords

We took a sector specific approach for health, agriculture, education, emergency nutrition, social protection, and water, sanitation, and hygiene (WASH) to search for nutrition-sensitive data activities. We first compiled a catchment of projects that were nutrition relevant from these sectors and then we used ND&IS data related keywords to further limit the dataset. Keywords used multiple languages, including English, French, and Spanish.

Nutrition-sensitive keywords	
acuicultura	food security
agrícola	food stamps
agricole	food subsidies
agricultura	food subsidy
agricultura sensible a la nutrición	food supplement
agricultura y nutrición	food system
agricultural	food voucher
agriculture	fortificación
agriculture and nutrition	fortificación de alimentos
agriculture et nutrition	fortification
agua potable	fortification des aliments
agua y el saneamiento	ganado
agua y saneamiento	garden
aide alimentaire	hambruna
alimentación directa	handwash
alimentación escolar	health information system
alimentación general	health management information system
alimentación infantil	higiene
alimentación suplementaria	hygiene
alimentación terapéutica	hygiène
alimentation	ingesta de alimentos
alimentation complémentaire	insécurité alimentaire
alimentation des enfants	inseguridad alimentaria
alimentation directe	jabón
alimentation nutritive	jardin
alimentation scolaire	jardín
alimentation thérapeutique	lavado de manos
alimento nutritivo	lavage des mains
alimentos de emergencia	livelihood
aliments nutritifs	livelihood and vulnerability
allocations	livestock
análisis integral de seguridad alimentaria y vulnerabilidad	malnutrition aiguë
analyse complète de la sécurité alimentaire et de la vulnérabilité	moyen de subsistance

aquaculture	moyen de subsistance et vulnérabilité
asistencia alimentaria	niñez temprana
assainissement	nourriture
banco de alimentos	nutrición y agricultura
banque alimentaire	nutrientes
bétail	nutrients
biofortificación	nutriments
Biofortification	nutrition and agriculture
blanket feeding	nutrition et agriculture
bons alimentaires	nutrition sensitive
cash transfer	nutrition sensitive agriculture
cfsva	nutrition supplement
cheptel	nutritional supplement
child feeding	nutrition-sensitive
comida nutritiva	nutrition-sensitive agriculture
comida por activo	nutritious food
comida por formación	pêcherie
complément alimentaire	pesquerías
comprehensive food security and vulnerability analysis	petite enfance
consommation de nourriture	programa de alimentación
consumo de alimentos	programme d'alimentation
consumo de comida	ración de alimentos
cupones de alimentos	ration alimentaire
desarrollo de la primera infancia	régime
desnutrición aguda	routine health information system
dévelopement de la petite enfance	saneamiento
DHIS2	sanitation
DHIS-2	savon
DHIS-II	school feeding
diet	sécurité alimentaire
dieta	seguridad alimentaria
dietary diversification	sensible a la nutrición
direct feeding	sistema alimentario
distribución de comida	soap
distribution de nourriture	social transfer
district health information system	subsidio alimentario
diversificación dietética	subsidios alimentarios
diversification alimentaire	subvention alimentaire
drinking water	subventions alimentaires
early childhood	suplemento alimenticio
early childhood development	suplemento nutricional
eau et assainissement	supplementary feeding

eau potable	sustento
émaciation	sustento y vulnerabilidad
emergency food	système alimentaire
famine	therapeutic feeding
feeding program	transferencia de efectivo
feeding programme	transferencia social
fisheries	transfert social
food aid	vale de comida
food bank	voucher
food consumption	vulnerabilidad
food distribution	vulnerabilidad y sustento
food for asset	vulnérabilité
food for training	vulnerability
food fortification	vulnerability and livelihood
food insecurity	wasting
food intake	water and sanitation
food ration	
**Data-related keywords (second layer)**	
collecte de données	monitoreo y evaluación
contrôle et évaluation	monitoreo y evaluación (M&E) de la nutrición
data	monitoreo y evaluación de la nutrición
data collection	monitoring and evaluation
datos	mSalud
demographic and health survey	mSanté
demographic health survey	multiple indicator cluster
demographic surveillance	niveau de vie
donnée	nutrition m&e
early warning system	recopilación de datos
education management information system	resource tracking
ehealth	revisión del gasto
encuesta	seguimiento de recursos
encuesta de Demografía y Salud	sistema de alerta temprana
encuesta nacional de ingresos y gastos de los hogares	sistema de información
encuesta SMART	sistema de información de gestión
enquête	sistema de información de gestión educativa
enquête démographique et sanitaire	smart survey
enquête SMART	standard de vie
enquête sur les dépenses des ménages	suivi des ressources
eSalud	suivi et évaluation
e-Santé	suivi nutritionnel
examen des dépenses	surveillance
expenditure review	surveillance de la nutrition

grappes à indicateurs multiples	surveillance démographique
household income and expenditure survey	survey
Indicadores Múltiples por Conglomerados	système d'alerte précoce
information system	système d'information
living standards measurement	système d'information et de gestion
m&e	système d'information et de gestion de l'éducation
management information system	système national d'information sanitaire
medición de los niveles de vida	vigilancia
mhealth	
