# Supplementary figures and images for: Landscape of official development assistance for nutrition data and information systems
Source: BMJ Glob Health. 2022 Mar 8;7(3):e007370. doi: 10.1136/bmjgh-2021-007370 (PMC8905917; doi:10.1136/bmjgh-2021-007370)

### Supplemental Figure 1: Screening Methodology

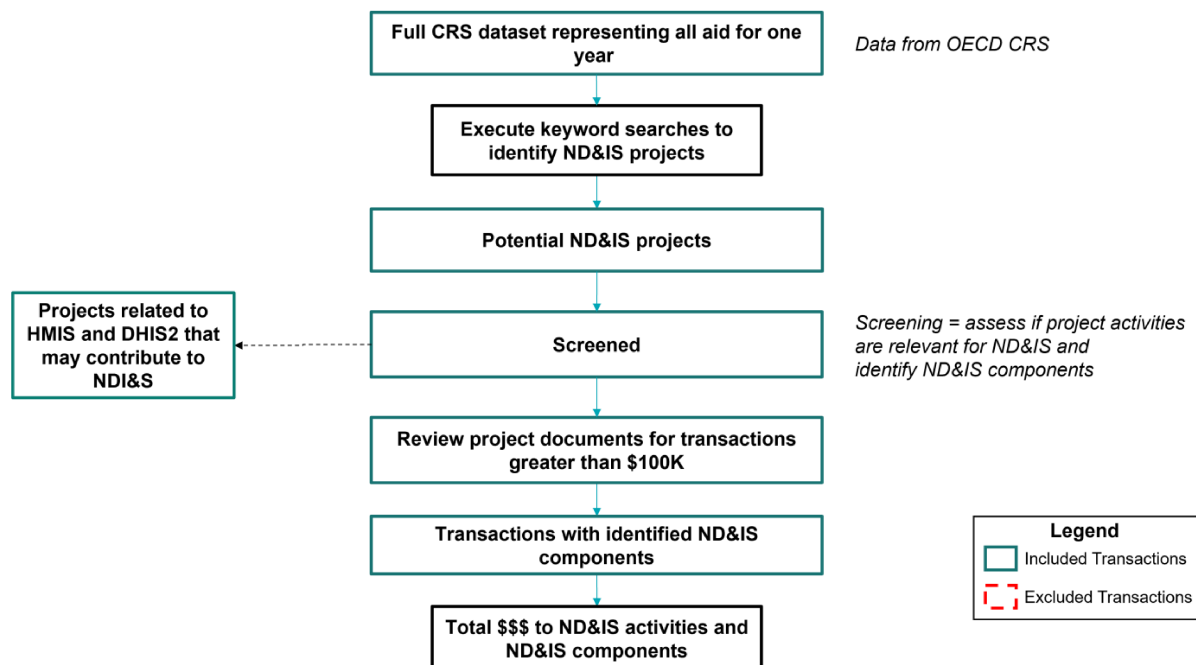

Supplement: Supplementary data [file bmjgh-2021-007370supp001.pdf]
