# Supplementary material for: Landscape of official development assistance for nutrition data and information systems
Source: BMJ Glob Health. 2022 Mar 8;7(3):e007370. doi: 10.1136/bmjgh-2021-007370 (PMC8905917; doi:10.1136/bmjgh-2021-007370)
Supplement: Supplementary data [file bmjgh-2021-007370supp003.pdf]

Supplemental Figure 2: Sensitivity Analysis for Upper Estimate of Total Spending Towards ND&IS in 2017-2019, millions USD

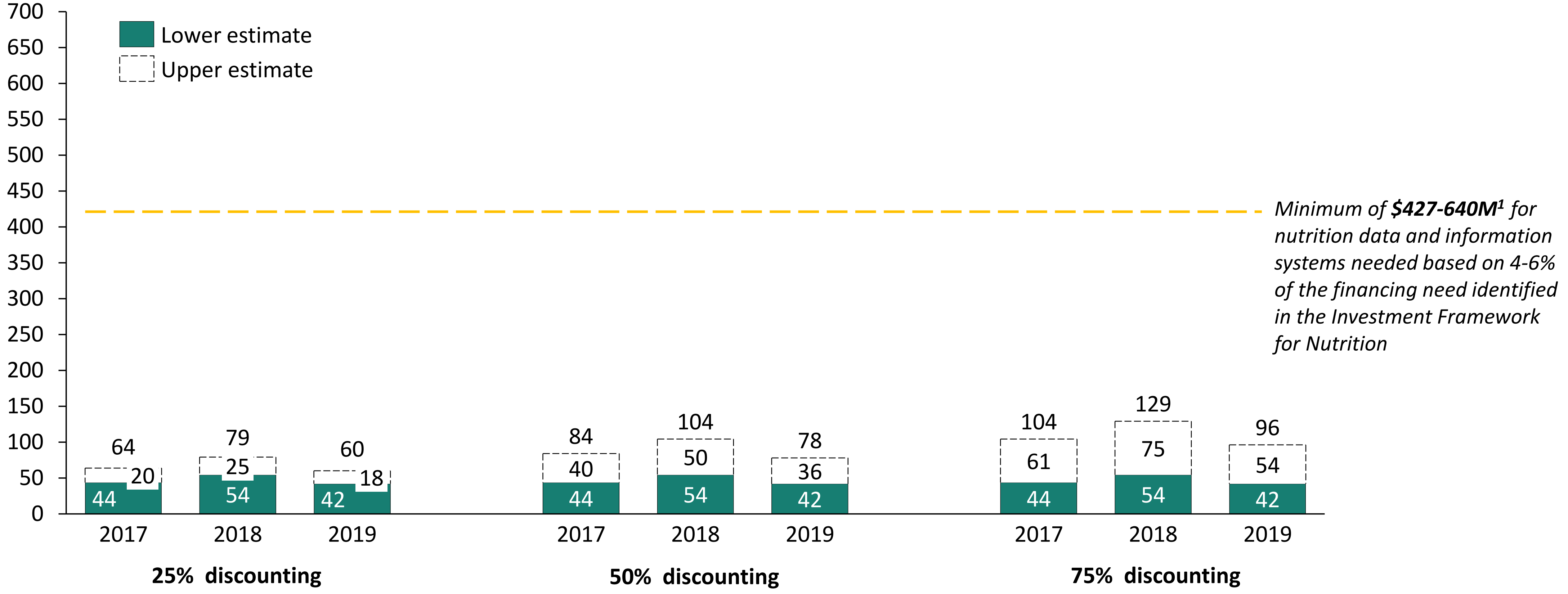

1. The Global Investment Framework for Nutrition estimates that $70b is needed from all sources over the next 10 years to reach the WHA targets for nutrition. Therefore, for one year, we assumed there is a need of $10.4b in financing for nutrition. We then took 98% of this number to account only for direct program costs. Next, we applied the 4-6% benchmark determined by the N4G financing working group to this estimate i.e., 98% of $10.4b which gave us the estimate of $427m to $640m per year needed for nutrition data related activities.
