# Supplementary material for: Landscape of official development assistance for nutrition data and information systems
Source: BMJ Glob Health. 2022 Mar 8;7(3):e007370. doi: 10.1136/bmjgh-2021-007370 (PMC8905917; doi:10.1136/bmjgh-2021-007370)
Supplement: Supplementary data [file bmjgh-2021-007370supp007.pdf]

Supplemental Table 4: Example projects captured under each ND&IS category

ND&IS Category	Type of Project Captured	Examples
**Global Initiatives**	Global public goods such as nutrition databases, reports, scorecards, or profiles	Global Nutrition Report, Access to Nutrition Index (ATNI), Global Open Data for Agriculture and Nutrition (GODAN), etc.
	Global level coalitions/partnerships that have ND&IS related activities	MQSUN+, Alive & Thrive, National Information Platforms for Nutrition (NIPN), etc.
**Routine Data Collection**	Projects focused on systems strengthening for routine information systems for nutrition	Strengthen the existing routine nutrition information systems to inform policies and programs in Burkina Faso; Strengthen Tanzania food and nutrition security information system
	Projects focused on establishing a nutrition surveillance system	National Food Security Nutritional Surveillance Project (FSNSP) to strengthen and institutionalize the nutritional surveillance system in Bangladesh
**Emergency Surveillance/Early Warning Systems (EWS)**	Projects related to Famine Early Warning Systems Network (FEWS NET)	FEWS NET technical support for web-based data capture, analysis, and visualization tools to better identify and report on famine threats
	Projects focused on establishing an emergency food security or nutrition surveillance/EWS system	Strengthen nutrition surveillance in drought-prone districts of Madagascar; Strengthen early warning systems for nutrition
**Periodic Data Collection**	Nationally representative multi-topic household surveys	Demographic & Health Survey, SMART Survey, Living Standards Measurement Study, Multiple Indicator Cluster Surveys, etc.
	Other types of large-scale surveys	Fortification Assessment Coverage Toolkit (FACT) surveys, Comprehensive Food Security & Vulnerability Analysis, Micronutrient and Food Consumption survey, etc.
**Country Capacity Building**	Global level coalitions/partnerships that include country level technical assistance for ND&IS	NIPN, MQSUN+, etc.
	Capacity building for routine information systems for nutrition	Technical assistance to improve nutrition data availability and use within routine data systems in Bangladesh; Improve capacity of local actors for nutritional surveillance
	Technical assistance and capacity building for surveys	Build capacity of governments to generate national data on fortification quality through FACT surveys; Build capacity to conduct the National Food and Nutrition Survey in Paraguay
	Projects related to training of national stakeholders to improve collection, usage, analysis, monitoring, reporting, and dissemination of nutrition related data	Build capacity to use the Food Insecurity Experience Scale (FIES) and the Prevalence of Undernourishment Data (PoU); Strengthen district capacity for monitoring of nutrition programs and services
	Nutrition measurement innovations	Support new methods for measuring micronutrient status; Develop a new integrated information and communication technology (ICT) tool for assessing food consumption
	Development or implementation of M&E	Implement, monitor, and evaluate multi-sectoral and multi-stakeholder national plans for nutrition

ND&IS Category	Type of Project Captured	Examples
Other	components/targets within national nutrition plans	
	Situation analyses	Conduct a situation analysis to assess resources, gaps, and priority needs for improved nutrition; Map health and nutrition activities across South Sudan to provide data for planning
	Develop, update, or review nutrition-related datasets	Support government in the review and technical update of Kenya Food Composition Tables; Develop and validate Food-Based Dietary Guidelines (FBDGs) for Ethiopia
